# Association of Vegetable, Fruit, and Okinawan Vegetable Consumption With Incident Stroke and Coronary Heart Disease

**DOI:** 10.2188/jea.JE20180130

**Published:** 2020-01-05

**Authors:** Takahiro Yoshizaki, Junko Ishihara, Ayaka Kotemori, Junpei Yamamoto, Yoshihiro Kokubo, Isao Saito, Hiroshi Yatsuya, Kazumasa Yamagishi, Norie Sawada, Motoki Iwasaki, Hiroyasu Iso, Shoichiro Tsugane, the JPHC Study Group

**Affiliations:** 1 Department of Food and Life Sciences, Faculty of Food and Nutritional Sciences, Toyo University, Gunma, Japan; 2 School of Life and Environmental Science, Department of Food and Life Science, Azabu University, Kanagawa, Japan; 3 Epidemiology and Prevention Group, Center for Public Health Sciences, National Cancer Center, Tokyo, Japan; 4 Department of Preventive Cardiology, National Cerebral and Cardiovascular Center, Osaka, Japan; 5 Department of Community Health Systems Nursing, Ehime Graduate School of Medicine, Ehime, Japan; 6 Department of Public Health, School of Medicine, Fujita Health University, Aichi, Japan; 7 Department of Public Health Medicine, Faculty of Medicine, University of Tsukuba, Ibaraki, Japan; 8 Public Health, Department of Social Medicine, Osaka University Graduate School of Medicine, Osaka, Japan

**Keywords:** cohort study, Okinawan vegetable, cardiovascular disease

## Abstract

**Background:**

Few studies have investigated the effects of Okinawan vegetable consumption on the risk of incident stroke and coronary heart disease. This study aimed to examine associations of vegetable, fruit, and Okinawan vegetable consumption with risk of incident stroke and coronary heart disease in the Japanese population of Okinawa.

**Methods:**

The study design was a prospective cohort study. During 1995–1998, a validated food frequency questionnaire was administered in two study areas to 16,498 participants aged 45–74 years. In 217,467 person-years of follow-up until the end of 2012, a total of 839 stroke cases and 197 coronary heart disease cases were identified.

**Results:**

No statistically significant association between total Okinawan vegetable consumption and risk of stroke and coronary heart disease was obtained: the multivariable adjusted hazard ratios for the highest versus lowest tertile of consumption were 1.09 (95% confidence interval, 0.93–1.29; *P for trend* = 0.289) in model 2. Total vegetable and fruit and specific Okinawan vegetable consumption were also not statistically significantly associated with risk of cardiovascular outcomes.

**Conclusions:**

This study demonstrated that consumption of total vegetable and fruit, total Okinawan vegetables, and specific Okinawan vegetables in Japanese residents of Okinawa was not associated with risk of incident stroke and coronary heart disease.

## INTRODUCTION

A third of all deaths worldwide result from cardiovascular disease (CVD).^1^ Large declines in mortality caused by CVD (especially heart disease) in developed countries, including Japan, have started to level off over the past 25 years.^1^ These declines have been linked to changes in sociodemographic factors, especially in regions with high sociodemographic status, and eating habits may be one of the factors related to such declines. A beneficial effect of fruit and vegetable consumption on the risk of coronary heart disease in Western populations and mortality from CVD in the Japanese population has been suggested^2^^,^^3^; however, in Japan, a recent large cohort study by Takachi et al showed no statistically significant association between vegetable consumption and risk of CVD.^4^

In the Okinawa Prefecture, which is located in the southwestern part of Japan and has a subtropical and oceanic climate, Okinawan vegetables, such as pak choi, leaf mustard, bitter gourd, Swiss chard, loofah, mugwort, and papaya, are commonly and traditionally eaten among Okinawans and are largely produced in Okinawa Prefecture. Okinawan vegetables contain large amounts of folate and fibre, which are inversely associated with higher homocysteine concentration, a potential risk factor for coronary heart disease and stroke.^5^^–^^7^ A higher dietary intake of folate has been associated with lower risk of incident stroke and mortality from stroke, coronary heart disease, and heart failure.^8^^–^^11^ Furthermore, Okinawan vegetables contain several functional substances that have been indicated to affect health in the molecular level. For example, bitter gourds have demonstrated antioxidant activities in both *in vivo* and *in vitro* experiments^12^ and can decrease low-density lipoprotein cholesterol and triglyceride levels, suppress visceral fat accumulation, and inhibit adipocyte hypertrophy in diet-induced obese rats and mice.^13^ Moreover, papaya extracts and papaya-associated phytochemicals possess anti-inflammatory and immunomodulatory properties.^14^ A trial study has reached the stage of verifying the effect of consuming typical Okinawan vegetable dishes on the number of circulating endothelial progenitor cells,^15^ which indicates a protective benefit in maintaining the integrity of vascular vessels. As Okinawan vegetables are rich in antioxidants and folic acid, their consumption can contribute to the prevention of CVD. However, no large prospective study has examined whether Okinawan vegetable consumption is associated with incident stroke and coronary heart disease.

Okinawans have a lower rate of age-adjusted mortality due to CVD compared with the rest of the Japanese population, although they have a higher prevalence of metabolic syndrome.^16^^,^^17^ The reasons are unclear, but Okinawa’s unique food, such as Okinawan vegetables, which are typically used in Okinawan dishes, may be a factor. Therefore, we aimed to elucidate associations of Okinawan vegetable consumption, in addition to total fruit and vegetable consumption, based on a validated comprehensive food frequency questionnaire (FFQ), with risk of incident stroke and coronary heart disease through a population-based prospective cohort study in Okinawa.

## MATERIALS AND METHODS

### Study population

The Japan Public Health Center-Based Prospective Study (JPHC Study) included two cohorts based on public health centre (PHC) areas: Cohort I (started in 1990, five PHC areas, participants aged 40–59 years) and Cohort II (started in 1993, six PHC areas, participants aged 40–69 years). The inhabitants in these 11 PHC areas were identified through population registries administered by the local municipalities. Details of the JPHC Study protocol have been shown in a previous study.^18^ The study protocol was approved by the institutional review board of Azabu University, Toyo University, and the National Cancer Center, Tokyo, Japan.

To identify associations between Okinawan vegetable consumption and risk of incident stroke and coronary heart disease, we limited the research area to the Okinawa Prefecture (two PHC areas: Chubu from Cohort I and Miyako from Cohort II), because the median value for Okinawan vegetable consumption was 41.7 g (5th–95th percentile, 6.8–161.1 g) in the Okinawa Prefecture, and 2.4 g (5th–95th percentile, 0.0–28.5 g) in other prefectures. Participants comprised 28,315 inhabitants (14,390 men and 13,925 women). A self-administrated questionnaire was distributed to all residents in the study areas for Cohorts I and II. The questionnaire included demographic characteristics, medical history, smoking, drinking, and a simplified version for dietary habits. Furthermore, a 5-year follow-up survey was conducted using a questionnaire in 1995 for Cohort I and in 1998 for Cohort II. This questionnaire was used as the starting point to assess dietary exposure in our study because it includes more comprehensive information on food intake frequency (ie, the FFQ) and demographic characteristics.

**Figure 1.**
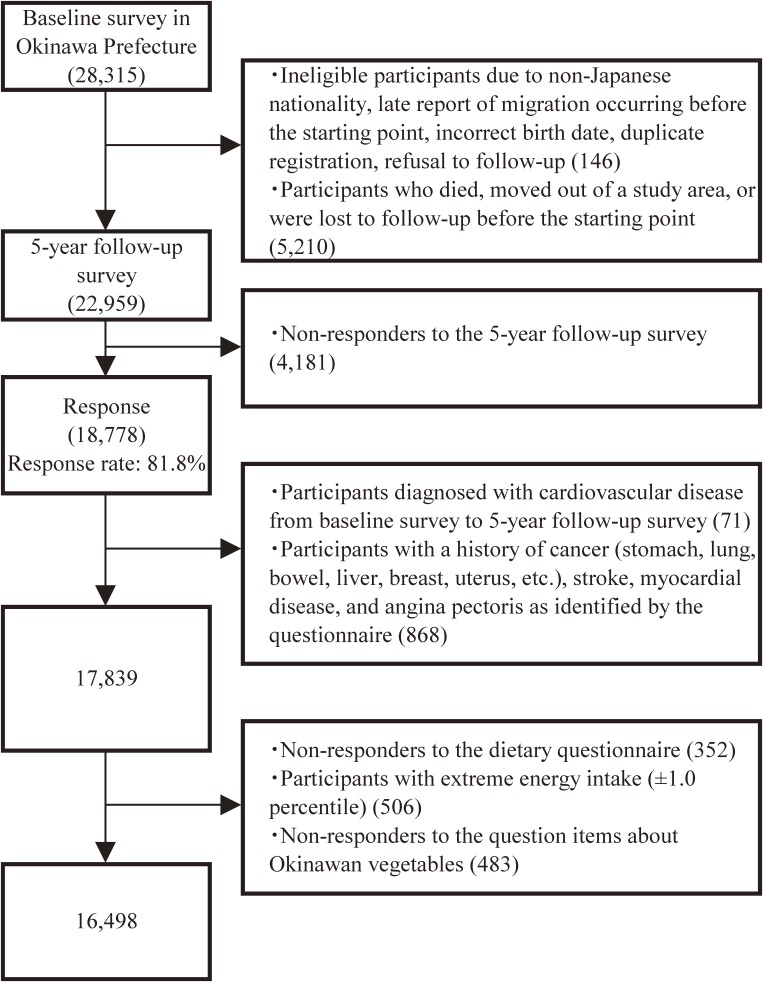
Flowchart of study participants in the Japan Public Health Center-Based Prospective Study on the association of total Okinawan vegetable consumption with incident stroke and coronary heart disease

We excluded participants with non-Japanese nationality, incorrect or late report of migration that occurred before the starting point, incorrect birth data, or duplicate registration, and those who refused follow-up, died, moved out of the study areas, or were lost to follow-up before the starting point. Finally, 22,959 participants were eligible for our study. Among them, 18,778 participants (8,941 men and 9,837 women) responded to the 5-year follow-up questionnaire, yielding a response rate of 81.8%, and were included in our study. Among the 18,778 respondents, we excluded participants who were diagnosed with CVD from the baseline survey to the 5-year follow-up survey (*n* = 71) and those who had a history of cancer (eg, stomach, lung, bowel, liver, breast, or uterus), stroke, myocardial disease, and angina pectoris as identified by the questionnaire (*n* = 868). Furthermore, 352 participants who did not complete the diet part of the questionnaire, 506 who reported extreme total energy intake (lower and upper 1.0 percentile for men and women: 767 and 5,475 kcal/day, and 617 and 4,619 kcal/day, respectively), and 483 who did not respond to the question items on Okinawan vegetables were excluded. Therefore, 16,498 participants (7,726 men and 8,772 women) were included in our statistical analysis (Figure 1).

### Assessment of food consumption and nutrients

The FFQ consisted of 138 food and beverage items with standard portions/units and 9 frequency categories. The FFQ asked about daily consumption of 7 Okinawan vegetables, 15 fruits, and 24 vegetables during the previous year (eTable 1). The Okinawan vegetable items were pak choi, leaf mustard, bitter gourd, Swiss chard, loofah, mugwort, and papaya (eTable 2). Because Okinawans eat green papaya (immature papaya) as a salad or stir-fried dish, we included papaya as an Okinawan vegetable item. The fruit items included 3 citrus fruits (mandarin orange, other oranges, and 100% orange juice) and 12 other fruits (apple, persimmon, strawberry, grapes, melon, watermelon, peach, pear, kiwi fruit, pineapple, banana, and 100% apple juice). The vegetable items comprised six pickled vegetables (Chinese radish, green leafy vegetables, plum, Chinese cabbage, cucumber, and eggplant), five cruciferous vegetables (cabbage, Chinese radish, komatsuna, broccoli, and Chinese cabbage), four green leafy vegetables (spinach, Chinese chives, garland chrysanthemum, and green pepper), four yellow vegetables (carrot, tomato, pumpkin, and tomato juice), and five other vegetables (onion, cucumber, bean sprouts, snap beans, and lettuce).

The 5-year follow-up questionnaire was based on nine frequency categories (‘almost never’ to ‘7 or more times per day’) for vegetables, fruits, and Okinawan vegetables, and nine frequency choices (‘almost never’ to ‘10 or more glasses per day’) for juices. Standard portion sizes were fixed for each food item as follows: small (50% smaller), medium (same as the standard), and large (50% larger). The amounts (g/day) of vegetables, fruits, and Okinawan vegetables consumed were calculated from the data obtained.

The FFQ for assessing vegetable and fruit consumption has been already validated in previous studies^19^^,^^20^: the deattenuated correlation coefficient between energy-adjusted consumption based on the FFQ and that based on 28-day (or 14-day for the Chubu PHC area) dietary records among subsamples of men and women in Okinawa Prefecture were 0.56 for fruits, 0.43 for vegetables, and 0.52 for total vegetable and fruit. The correlation coefficients for the reproducibility of the FFQ administered 1 year apart for men and women were 0.42 for fruits, 0.49 for vegetables, and 0.40 for total vegetable and fruit consumption. Meanwhile, the validity of the FFQ for assessment of total Okinawan vegetable consumption and specific Okinawan vegetable consumption was as follows: the deattenuated correlation coefficients for men and women were 0.14 for total Okinawan vegetable, 0.30 for pak choi, 0.025 for leaf mustard, 0.16 for bitter gourd, 0.47 for Swiss chard, 0.37 for loofah, 0.38 for mugwort, and 0.37 for papaya. Moreover, the correlation coefficients for the reproducibility of the FFQ for men and women were 0.47 for total Okinawan vegetable, 0.45 for pak choi, 0.67 for leaf mustard, 0.41 for bitter gourd, 0.45 for Swiss chard, 0.50 for loofah, 0.47 for mugwort, and 0.42 for papaya.

### Follow-up survey

Participants in the Chubu PHC area (Cohort I) and Miyako PHC area (Cohort II) were followed from 1995 and 1998 until December 31 of 2009 and 2012, respectively. Information about changes in residence status was obtained annually from data on moving out, which are identified by residency registration inside and outside the study areas. All mortality data for participants in the residential registry are forwarded to the Ministry of Health, Labour and Welfare and are coded for inclusion in the National Vital Statistics. As residency and death registrations are required by the Basic Resident Registration Law and Family Registration Law, respectively, data from each registration are thought to be complete. Based on both data, we confirmed that 3,494 subjects (18.6%) died, 676 (3.6%) moved out of the study area, and 5 (0.03%) were lost to follow-up during the follow-up period in our study.

### Ascertainment of stroke and coronary heart disease incidence

A total of 35 hospitals formed the register of events within the Chubu and Miyako PHC areas. All were major hospitals with the capability of treating patients with acute coronary heart disease, stroke, or cancer events. The medical records were reviewed by hospital or PHC physicians in each registered major local hospital in each PHC area.^21^^,^^22^ Diagnoses of stroke using computer tomography scan and/or magnetic resonance imaging according to the criteria of the National Survey of Stroke^23^ and diagnoses of myocardial infarction according to the criteria of the MONICA project^24^ were confirmed, for all cases, and these medical data were extracted onto cohort-specific registration forms. Stroke cases were classified according to subtype (ie, intraparenchymal haemorrhage, subarachnoid haemorrhage, or ischaemic stroke). CVD cases with a death certificate or self-report only, without confirmation via medical records, were excluded from outcome events. CVD was defined as myocardial infarction or stroke, whichever occurred first. We confirmed 839 stroke cases and 197 myocardial infarction cases among the 16,498 subjects by December 31 of 2009 and 2012. The rate of implementation for computed tomography scans, magnetic resonance imaging, and/or autopsy findings in Okinawa Prefecture (99.3% in the Chubu area and 98.7% in the Miyako area) did not differ from that in other PHC areas (98.4%) of the JPHC Study.

For analysis, only first-ever CVD events during the follow-up were included; recurrent events were excluded. Person-years of follow-up were calculated for each participant from baseline to the date of death, diagnosis (earlier event for coronary heart disease and stroke), emigration from the study area, or end of the follow-up period (December 31 of 2009 and 2012), whichever occurred first. Participants lost to follow-up were censored at the last confirmed date of their presence in the study area. A total of 217,467 person-years for the CVD analysis was accrued.

### Statistical analysis

Participants in each group were separated into tertiles (T1–T3) according to consumption, with the lowest consumption category as the reference. Hazard ratios and 95% confidence intervals were calculated for categories of energy-adjusted consumption of vegetables, fruits, total Okinawan vegetables, and specific Okinawan vegetables, using Cox proportional hazards models according to the SAS PHREG Procedure (SAS Institute, Inc., Cary, NC, USA). Multivariate Cox regression models with a covariate adjustment approach for total vegetable and fruit, total vegetable, total fruit, total Okinawan vegetable, and specific Okinawan vegetable consumption were used: model l included age at baseline, study area, and sex; model 2 included model 1 plus covariates, including alcohol intake (0, 1–150, 151–300, 301–450, and ≥451 g/week, or missing), cigarette smoking status (current [<20 or ≥20 cigarettes/day], never, former, or missing), type of job (blue-collar, white-collar, other, or missing), self-reported perceived mental stress (low, medium, high, or missing), quartiles of body mass index (BMI), metabolic equivalent task-hours per day (METs), and dietary-related factors (ie, quartiles of energy intake and energy-adjusted dietary consumption of fish, meat, and sodium); model 3 included model 2 plus mediator variables, including past history of diabetes (yes, no, or missing), treatment of hypertension (yes, no, missing), and treatment of hypercholesterolaemia (yes, no, missing) based on either self-reported past history of diabetes or hypoglycaemic agent use, antihypertensive agent use, and hypercholesterolaemia agent use, respectively. Quartiles of energy-adjusted dietary consumption of total fruits were added to model 2 in the multivariate analysis for total vegetables; quartiles of total vegetable consumption were added for total fruits; and quartiles of vegetable (except for Okinawan vegetables) and fruit (except for papaya) consumption were added for total Okinawan vegetables and specific Okinawan vegetables. The missing data of covariates was adjusted using a dummy variable. Each food group consumption was adjusted by total energy intake using the residual method.^25^ Two-tailed *P* values of <0.05 were considered statistically significant.

## RESULTS

Consumption of total vegetables, fruits, and Okinawan vegetables ranged from a median of 187.3 to 544.4 g/day (lowest to highest tertile). Total Okinawan vegetable consumption by the participants ranged from a median of 17.9 to 94.3 g/day (lowest to highest tertile). Associations between total Okinawan vegetable consumption and demographic characteristics of participants according to tertiles are shown in Table 1. In men and women, higher Okinawan vegetable consumption was significantly associated with higher age, METs, and intakes of energy, fish, meat, fibre, sodium, calcium, total vegetables and fruits, total vegetables, and total fruits (*P* < 0.05). Total Okinawan vegetable consumption was significantly associated with sex, study area, smoking habit, treatment of hypertension, treatment of hypercholesterolemia, past history of diabetes, drinking habit, type of work, and self-reported perceived mental stress (*P* < 0.05; Table 1). Individuals with higher Okinawan vegetable consumption were less likely to be male, ever-smokers, or heavy drinkers, and they were more likely to be receiving treatment for hypertension or hypercholesterolemia, or have a past history of diabetes and blue-collar job. Meanwhile, BMI, employment status, and sleep duration were not significantly associated with total Okinawan vegetable consumption.

**Table 1.** Baseline characteristics of the study participants

		Tertile of total Okinawan vegetable consumption						*P* values^a^

		Number	Lowest (T1)	Number	Middle (T2)	Number	Highest (T3)	
Age	years	5,499	56 (8)	5,500	57 (8)	5,499	58 (8)	<0.001
Body mass index	kg/m^2^	5,401	24.4 (3.3)	5,401	24.4 (3.2)	5,409	24.5 (3.3)	0.086
Sex								
Women			2,608 (47.4)		3,005 (54.6)		3,159 (57.4)	<0.001
Men			2,891 (52.6)		2,495 (45.4)		2,340 (42.6)	
Study area								
Chubu area			3,289 (59.8)		3,044 (55.3)		2,809 (51.1)	<0.001
Miyako area			2,210 (40.2)		2,456 (44.7)		2,690 (48.9)	
Smoking habit								
Never			3,652 (70.3)		3,896 (74.4)		4,103 (78.1)	<0.001
Former			396 (7.6)		387 (7.4)		365 (6.9)	
1–20 cigarettes			306 (5.9)		305 (5.8)		266 (5.1)	
>20 cigarettes			839 (16.2)		647 (12.4)		521 (9.9)	
Drinking habit								
Never			3,063 (55.7)		3,382 (61.5)		3,705 (67.4)	<0.001
1–150 mL			190 (3.5)		205 (3.7)		215 (3.9)	
150–300 mL			172 (3.1)		194 (3.5)		198 (3.6)	
300–450 mL			104 (1.9)		108 (2.0)		103 (1.9)	
>450 mL			1,970 (35.8)		1,611 (29.3)		1,278 (23.2)	
Treatment of hypertension								
No			4,358 (80.3)		4,343 (79.9)		4,253 (78.3)	0.023
Yes			1,066 (19.7)		1,093 (20.1)		1,177 (21.7)	
Treatment of hypercholesterolaemia								
No			5,276 (97.6)		5,262 (97.1)		5,239 (96.7)	0.027
Yes			132 (2.4)		158 (2.9)		179 (3.3)	
Past history of diabetes								
No			5,249 (95.5)		5,220 (94.9)		5,190 (94.4)	0.038
Yes			250 (4.5)		280 (5.1)		309 (5.6)	
Type of work								
Blue-collar			949 (17.9)		958 (18.1)		1,074 (20.2)	<0.001
White-collar			3,899 (73.5)		3,900 (73.5)		3,767 (70.8)	
Other			456 (8.6)		445 (8.4)		480 (9.0)	
Employment								
Employed			4,911 (92.6)		4,937 (93.1)		4,930 (92.7)	0.544
Non-employed			393 (7.4)		366 (6.9)		391 (7.3)	
Self-reported perceived mental stress								
Low			1,228 (22.8)		1,244 (22.9)		1,362 (25.2)	<0.001
Middle			3,353 (62.1)		3,461 (63.7)		3,241 (60.0)	
High			816 (15.1)		727 (13.4)		797 (14.8)	
Sleep duration								
<6 hours			362 (7.2)		338 (6.6)		339 (6.7)	0.513
≥6 hours			5,042 (93.3)		5,099 (93.8)		5,092 (93.8)	
METs/week		4,567	33.0 (6.1)	4,647	33.5 (6.1)	4,623	33.8 (6.2)	<0.001

Energy intake	kcal/day	5,499	1913 (734)	5,500	1933 (695)	5,499	1872 (660)	0.002
Dietary intakes^b^		5,499		5,500		5,499		
Fish	g/day		55.5 (41.7)		64.7 (42.1)		67.6 (43.3)	<0.001
Meat	g/day		81.8 (50.9)		81.1 (46.9)		71.3 (45.1)	<0.001
Fibre	g/day		8.6 (3.4)		10.9 (3.4)		14.5 (4.7)	<0.001
Sodium	mg/day		3973 (1359)		4482 (6664)		5133 (7783)	<0.001
Calcium	mg/day		428 (238)		464 (213)		530 (208)	<0.001
Total vegetables and fruits	g/day		254 (153)		351 (159)		508 (206)	<0.001
Vegetables	g/day		144.2 (92.1)		211.0 (97.9)		334.9 (154.0)	<0.001
Fruits	g/day		110.4 (106.9)		140.5 (111.2)		173.6 (123.6)	<0.001
Total Okinawan vegetables	g/day		17.3 (7.7)		45.0 (9.5)		114.9 (69.5)	<0.001
Pak choi	g/day		1.8 (2.7)		4.8 (5.8)		11.9 (16.1)	<0.001
Leaf mustard	g/day		1.7 (2.4)		4.5 (5.2)		11.8 (16.1)	<0.001
Bitter gourd	g/day		6.9 (5.3)		18.3 (10.5)		43.8 (36.3)	<0.001
Swiss chard	g/day		0.9 (1.9)		2.5 (4.6)		8.9 (19.2)	<0.001
Loofah	g/day		3.1 (3.1)		8.7 (7.2)		24.9 (26.5)	<0.001
Mugwort	g/day		0.2 (0.4)		0.3 (0.5)		0.5 (1.0)	<0.001
Papaya	g/day		2.6 (2.9)		5.5 (6.3)		11.4 (19.0)	<0.001

MET, metabolic equivalent task-hours per day.

Values are expressed as mean (standard deviation) or number (%).

^a^Determined using the one-way analysis of variance with polynomial contrasts or the chi-square test.

^b^Dietary intakes were energy-adjusted using the nutrient residual method.

The results of age-, sex-, and study area-adjusted (model 1) and multivariate-adjusted (model 2 and model 3) hazard ratios of CVD outcomes according to consumption of total vegetables and fruits, total vegetables, and total fruits are shown in Table 2. Significant inverse associations were found between total fruit consumption and risk of total CVD, total stroke, and ischaemic stroke in model 1. However, when we added other confounding variables in model 2, these significant results disappeared. Consumption of total vegetables and fruits was not significantly associated with risk of CVD outcomes in any model, while higher consumption of total vegetable was statistically significantly associated with the increased risk of total CVD, total stroke, and intraparenchymal haemorrhage in model 2. When we added mediator variables in model 3, the statistically significant associations between the consumption of total vegetable and the risks of total CVD and total stroke disappeared. Table 3 shows the associations between total and specific Okinawan vegetable consumption and CVD outcomes. As a result, any total and specific Okinawan vegetable consumption was not significantly associated with risk of CVD outcomes in model 2. In addition, these results of an association of total vegetable and fruit, total vegetable, total fruit, total Okinawan vegetable, and specific Okinawan vegetable consumption with risk of CVD outcomes were also confirmed when the data was divided by sex-specific tertiles (eTable 3 and eTable 4); the null association did not change.

**Table 2.** Age-, sex-, and study area-adjusted and multivariate-adjusted hazard ratios and 95% confidence intervals of incident cardiovascular outcomes according to tertiles of consumption of total vegetables and fruits, total vegetables, and total fruits

		Lowest (T1)	Middle (T2)		Highest (T3)		*P for trend*
		
		Ref.	HR	(95% CI)	HR	(95% CI)	
**Total vegetable and fruit consumption**^e^							
Number of participants		5,499	5,500		5,499		—
Median intake, g/day		187.3	337.0		544.4		—
Person-years		71,813	73,062		72,592		—
Cardiovascular disease, total	Number of cases	373	314		349		
	Model 1^a^	1.0	**0.83**	**(0.71–0.96)**	0.92	(0.79–1.07)	0.264
	Model 2^b^	1.0	0.90	(0.77–1.05)	1.06	(0.90–1.25)	0.518
	Model 3^c^	1.0	0.90	(0.77–1.06)	1.05	(0.89–1.24)	0.584
Stroke, total	Number of cases	300	255		284		
	Model 1^a^	1.0	**0.83**	**(0.70–0.98)**	0.91	(0.77–1.07)	0.254
	Model 2^b^	1.0	0.91	(0.76–1.08)	1.07	(0.89–1.28)	0.508
	Model 3^c^	1.0	0.91	(0.77–1.09)	1.06	(0.88–1.27)	0.571
Intraparenchymal haemorrhage	Number of cases	100	87		104		
	Model 1^a^	1.0	0.85	(0.64–1.14)	1.01	(0.76–1.33)	0.950
	Model 2^b^	1.0	0.99	(0.73–1.34)	1.31	(0.96–1.79)	0.091
	Model 3^c^	1.0	0.99	(0.73–1.34)	1.30	(0.95–1.78)	0.097
Ischaemic stroke	Number of cases	178	154		154		
	Model 1^a^	1.0	0.84	(0.68–1.05)	0.84	(0.67–1.04)	0.110
	Model 2^b^	1.0	0.89	(0.71–1.11)	0.91	(0.71–1.16)	0.432
	Model 3^c^	1.0	0.90	(0.72–1.12)	0.90	(0.71–1.15)	0.388
Coronary heart disease (myocardial infarction or sudden cardiac death)	Number of cases	73	59		65		
	Model 1^a^	1.0	0.83	(0.59–1.17)	0.97	(0.69–1.36)	0.830
	Model 2^b^	1.0	0.87	(0.61–1.24)	1.05	(0.72–1.52)	0.836
	Model 3^c^	1.0	0.86	(0.60–1.23)	1.04	(0.71–1.51)	0.875

**Total vegetable consumption**^e^							
Number of participants		5,499	5,500		5,499		—
Median intake, g/day		109.9	203.1		337.9		—
Person-years		72,053	72,831		72,583		—
Cardiovascular disease, total	Number of cases	353	310		373		
	Model 1^a^	1.0	0.88	(0.75–1.02)	1.00	(0.86–1.16)	0.972
	Model 2^b,d^	1.0	0.98	(0.83–1.15)	**1.19**	**(1.00–1.41)**	**0.047**
	Model 3^c^	1.0	0.97	(0.82–1.14)	1.15	(0.97–1.37)	0.097
Stroke, total	Number of cases	289	242		308		
	Model 1^a^	1.0	**0.83**	**(0.70–0.99)**	0.99	(0.85–1.17)	0.978
	Model 2^b,d^	1.0	0.94	(0.79–1.13)	**1.22**	**(1.01–1.48)**	**0.037**
	Model 3^c^	1.0	0.93	(0.78–1.12)	1.18	(0.98–1.43)	0.080
Intraparenchymal haemorrhage	Number of cases	94	86		111		
	Model 1^a^	1.0	0.91	(0.68–1.23)	1.12	(0.85–1.48)	0.402
	Model 2^b,d^	1.0	1.12	(0.82–1.52)	**1.56**	**(1.13–2.16)**	**0.007**
	Model 3^c^	1.0	1.11	(0.82–1.51)	**1.54**	**(1.12–2.13)**	**0.009**
Ischaemic stroke	Number of cases	175	137		174		
	Model 1^a^	1.0	**0.78**	**(0.62–0.97)**	0.93	(0.75–1.14)	0.483
	Model 2^b,d^	1.0	0.85	(0.67–1.08)	1.06	(0.83–1.35)	0.645
	Model 3^c^	1.0	0.84	(0.66–1.06)	1.01	(0.79–1.30)	0.907
Coronary heart disease (myocardial infarction or sudden cardiac death)	Number of cases	64	68		65		
	Model 1^a^	1.0	1.08	(0.77–1.52)	1.02	(0.72–1.45)	0.894
	Model 2^b,d^	1.0	1.12	(0.78–1.60)	1.07	(0.72–1.59)	0.746
	Model 3^c^	1.0	1.12	(0.78–1.60)	1.07	(0.72–1.60)	0.739

**Total fruit consumption**^e^							
Number of participants		5,499	5,500		5,499		—
Median intake, g/day		39.3	116.9		229.9		—
Person-years		71,710	72,906		72,851		—
Cardiovascular disease, total	Number of cases	387	332		317		
	Model 1^a^	1.0	0.88	(0.76–1.02)	0.86	(0.74–1.00)	**0.049**
	Model 2^b,d^	1.0	0.92	(0.79–1.08)	0.91	(0.77–1.06)	0.227
	Model 3^c^	1.0	0.94	(0.81–1.10)	0.93	(0.79–1.09)	0.377
Stroke, total	Number of cases	317	273		249		
	Model 1^a^	1.0	0.87	(0.74–1.02)	**0.81**	**(0.69–0.96)**	**0.012**
	Model 2^b,d^	1.0	0.92	(0.78–1.09)	0.86	(0.72–1.03)	0.103
	Model 3^c^	1.0	0.95	(0.80–1.12)	0.89	(0.74–1.06)	0.191
Intraparenchymal haemorrhage	Number of cases	114	90		87		
	Model 1^a^	1.0	0.79	(0.60–1.05)	0.78	(0.59–1.03)	0.074
	Model 2^b,d^	1.0	0.81	(0.61–1.08)	0.82	(0.61–1.11)	0.182
	Model 3^c^	1.0	0.82	(0.61–1.09)	0.83	(0.61–1.12)	0.205
Ischaemic stroke	Number of cases	185	165		136		
	Model 1^a^	1.0	0.91	(0.74–1.13)	**0.77**	**(0.62–0.97)**	**0.025**
	Model 2^b,d^	1.0	0.98	(0.79–1.22)	0.82	(0.64–1.03)	0.096
	Model 3^c^	1.0	1.01	(0.82–1.26)	0.85	(0.67–1.07)	0.184
Coronary heart disease (myocardial infarction or sudden cardiac death)	Number of cases	70	59		68		
	Model 1^a^	1.0	0.91	(0.64–1.28)	1.12	(0.80–1.57)	0.520
	Model 2^b,d^	1.0	0.93	(0.65–1.32)	1.14	(0.80–1.63)	0.464
	Model 3^c^	1.0	0.93	(0.65–1.33)	1.15	(0.80–1.63)	0.462

CI, confidence interval; HR, hazard ratio.

^a^Model 1 was adjusted by age, sex, and study area.

^b^Model 2 was adjusted by variables in model 1 plus alcohol intake (0, 1–150, 151–300, 301–450, and ≥451 g/week, or missing), cigarette smoking status (current [<20 or ≥20 cigarettes/day], never, former, or missing), type of work (blue-collar, white-collar, other, or missing), self-reported perceived mental stress (low, medium, high, or missing), quartiles of body mass index, metabolic equivalent task-hours per day, quartiles of energy intake, and energy-adjusted dietary consumption of fish, meat, and sodium.

^c^Model 3 was adjusted by variables in model 2 plus past history of diabetes (yes, no, or missing), treatment of hypertension (yes, no, or missing), and treatment of hypercholesterolaemia (yes, no, or missing).

^d^Quartiles of energy-adjusted dietary consumption of total fruits were added to model 2 in the multivariate analysis for total vegetables, and quartiles of total vegetable consumption were added for total fruits.

^e^Vegetable and fruit consumption was energy-adjusted by the nutrient residual method.

**Table 3.** Age-, sex-, and study area-adjusted and multivariate-adjusted hazard ratios and 95% confidence intervals of incident cardiovascular outcomes according to tertiles of total and specific Okinawan vegetable consumption

			Lowest (T1)	Middle (T2)		Highest (T3)		*P for trend*
		
			Ref.	HR	(95% CI)	HR	(95% CI)	
**Total Okinawan vegetable consumption**^d^								
	Number of participants		5,499	5,500		5,499		—
	Median intake, g/day		17.9	44.2		94.3		—
	Person-years		72,034	72,708		72,725		—
	Cardiovascular disease, total	Number of cases	352	322		362		
		Model 1^a^	1.0	0.91	(0.78–1.06)	0.99	(0.86–1.15)	0.927
		Model 2^b^	1.0	0.97	(0.83–1.14)	1.09	(0.93–1.29)	0.289
		Model 3^c^	1.0	0.96	(0.82–1.13)	1.07	(0.91–1.27)	0.403
	Stroke, total	Number of cases	283	260		296		
		Model 1^a^	1.0	0.92	(0.77–1.08)	1.01	(0.86–1.19)	0.876
		Model 2^b^	1.0	1.00	(0.84–1.19)	1.16	(0.96–1.39)	0.131
		Model 3^c^	1.0	0.99	(0.83–1.18)	1.13	(0.94–1.36)	0.203
	Intraparenchymal haemorrhage	Number of cases	101	86		104		
		Model 1^a^	1.0	0.86	(0.64–1.14)	1.02	(0.77–1.34)	0.898
		Model 2^b^	1.0	0.93	(0.69–1.25)	1.15	(0.84–1.57)	0.401
		Model 3^c^	1.0	0.93	(0.69–1.26)	1.14	(0.83–1.57)	0.411
	Ischaemic stroke	Number of cases	163	154		169		
		Model 1^a^	1.0	0.94	(0.76–1.17)	1.00	(0.80–1.24)	0.981
		Model 2^b^	1.0	1.02	(0.81–1.29)	1.14	(0.89–1.46)	0.296
		Model 3^c^	1.0	1.01	(0.80–1.27)	1.11	(0.86–1.41)	0.426
	Coronary heart disease (myocardial infarction or sudden cardiac death)	Number of cases	69	62		66		
		Model 1^a^	1.0	0.89	(0.63–1.26)	0.91	(0.65–1.28)	0.591
		Model 2^b^	1.0	0.88	(0.61–1.25)	0.89	(0.61–1.30)	0.549
		Model 3^c^	1.0	0.88	(0.62–1.26)	0.90	(0.62–1.31)	0.570

**Specific Okinawan vegetable consumption**^d^								
Pak choi	Median intake, g/day		0.0	2.6		11.4		—
	Cardiovascular disease, total	Model 3^c^	1.0	1.15	(0.99–1.33)	1.02	(0.87–1.20)	0.738
Leaf mustard	Median intake, g/day		0.0	2.6		10.6		—
	Cardiovascular disease, total	Model 3^c^	1.0	1.15	(0.99–1.34)	1.05	(0.89–1.24)	0.526
Bitter gourd	Median intake, g/day		4.4	15.6		39.2		—
	Cardiovascular disease, total	Model 3^c^	1.0	0.88	(0.75–1.03)	0.97	(0.83–1.14)	0.724
Swiss chard	Median intake, g/day		0.0	1.8		5.1		—
	Cardiovascular disease, total	Model 3^c^	1.0	1.06	(0.83–1.36)	1.06	(0.93–1.22)	0.374
Loofah	Median intake, g/day		1.7	5.6		22.1		—
	Cardiovascular disease, total	Model 3^c^	1.0	1.01	(0.87–1.18)	1.12	(0.95–1.31)	0.174
Mugwort	Median intake, g/day		0.0	0.2		0.5		—
	Cardiovascular disease, total	Model 3^c^	1.0	1.02	(0.87–1.19)	1.04	(0.89–1.22)	0.589
Papaya	Median intake, g/day		0.0	3.3		10.7		—
	Cardiovascular disease, total	Model 3^c^	1.0	0.94	(0.81–1.10)	1.07	(0.91–1.24)	0.420

CI, confidence interval; HR, hazard ratio.

^a^Model 1 was adjusted by age, sex, and study area.

^b^Model 2 was adjusted by variables in model 1 plus alcohol intake (0, 1–150, 151–300, 301–450, and ≥451 g/week, or missing), cigarette smoking status (current [<20 or ≥20 cigarettes/day], never, former, or missing), type of work (blue-collar, white-collar, other, or missing), self-reported perceived mental stress (low, medium, high, or missing), quartiles of body mass index, metabolic equivalent task-hours per day, quartiles of energy intake, and energy-adjusted dietary consumption of fish, meat, vegetable (except for Okinawan vegetables), fruit (except for papaya), and sodium.

^c^Model 3 was adjusted by variables in model 2 plus past history of diabetes (yes, no, or missing), treatment of hypertension (yes, no, or missing), and treatment of hypercholestaerolemia (yes, no, or missing).

^d^Total and specific Okinawan vegetable consumption was energy-adjusted using the nutrient residual method.

Stratified analyses according to age (<65 or ≥65 years), BMI (<18.5, 18.5–24.9, or ≥25 kg/m^2^), cigarette smoking status (smoker or non-smoker), alcohol intake (<150 or ≥150 g ethanol/week), meat consumption (<median or ≥median), and processed meat consumption (<median or ≥median), such as ham, Vienna sausage, bacon, and luncheon meat, showed that statistically significant associations between higher consumption of total vegetable and fruit, total vegetable, and total Okinawan vegetable and increased risk of CVD outcomes were more remarkable among those aged ≥65 years old, those with normal BMI (18.5–24.9 kg/m^2^), smokers, those with a higher consumption of meat, and those with a higher consumption of processed meat (data not shown).

## DISCUSSION

Our study aimed to elucidate associations between the consumption of total vegetables and fruits, total vegetables, total fruits, and total Okinawan vegetables, based on a validated comprehensive FFQ, with risk of CVD outcomes in a prospective cohort design. Multivariate Cox regression revealed that consumption of total vegetables and fruits, total vegetables, total Okinawan vegetables, and specific Okinawan vegetables was not significantly associated with risk of total CVD (*P* for trend > 0.05). Thus, total and specific Okinawan vegetable consumption and total vegetable and fruit consumption were not associated with risk of CVD outcomes, such as total stroke and coronary heart disease, in the population of Okinawa Prefecture.

Okinawan vegetables contain a large amount of folate. In our study, about half of the distribution of total folate intake could be explained by the distribution of total Okinawan vegetable consumption (data not shown). Folate decreases homocysteine level,^6^ which may explain why the plasma homocysteine levels of Okinawans are among the lowest in the world.^26^ Since higher blood homocysteine concentrations may be a potential risk factor for coronary heart disease and stroke,^6^ there is a high probability that Okinawan vegetable consumption can prevent CVD outcomes. In a prospective cohort study, Cui et al have also shown that high dietary intake of folate was associated with reduced risk of mortality from stroke, coronary heart disease, and heart failure among the Japanese people.^9^ However, no human study has been conducted on the direct association between Okinawan vegetable consumption and risk of CVD outcomes in a population-based cohort. Therefore, to the best of our knowledge, this is the first study to examine associations between Okinawan vegetable consumption and risk of CVD outcomes and to indicate that a significant inverse association could not be obtained—results that were contrary to our hypothesis. The reason why we found no association between the consumption of Okinawan vegetables and risk of CVD is unknown. However, it may be possible that the narrow range of Okinawan vegetables is a potential cause. In previous JPHC study, we found that folate and CVD were not statistically significantly associated because the range of dietary intake among the cohort population was narrow with a higher range (290 and 436 microgram/day for quintile 1 and 5, respectively) compared to that of the Western population.^27^ Likewise, it is plausible that even the reference group consumed a sufficient amount of folate from Okinawan vegetables to prevent CVD in this population, which resulted in a null association. In addition, it should be noted that both the sample size and number of cases were small.

Our definition of Okinawan vegetables needs careful consideration when interpreting the results, because there are other kinds of foods that are eaten as Okinawan vegetables in the Okinawa Prefecture (eg, Chinese preserving melon and Okinawan shallot) and we could not cover all of them in the FFQ. In addition, low validity of the FFQ for estimation of Okinawan vegetables, especially for leaf mustard, may have caused attenuation for the association between Okinawan vegetable consumption and risk of CVD. Therefore, although the seven Okinawan vegetable items may not be sufficient to classify total Okinawan vegetable consumption, not all specific Okinawan vegetables had low validity.

Our finding that there was no association between vegetable and fruit consumption and risk of CVD is not consistent with previous observations of cohort studies in European and Japanese populations.^2^^–^^4^ The follow-up period of our research (13.2 years) was relatively long; however, due to the fact that the research population was limited to Okinawa Prefecture, the number of CVD cases was lower than that in other studies. Therefore, although it may have been the optimal population to clarify the association between Okinawan vegetables and the risk of CVD at this time, care should be taken when interpreting the result that there was no association between vegetable and fruit consumption and the risk of CVD in our study. A statistically significant association between total vegetable consumption and intraparenchymal haemorrhage was found probably due to unmeasured confounders or by chance, though we do not have a good explanation.

We regarded past history of diabetes, treatment of hypertension, and treatment of hypercholesterolemia as mediators, but we further confirmed the adjusted results because these variables may be confounders as well as mediators on the causal pathway. However, the null associations of the consumption of total Okinawan vegetables and specific Okinawan vegetables with risks of CVD outcomes did not change.

This study has several limitations. First, the use of an FFQ, even if it was partially validated by 28-day dietary records, can inevitably lead to misclassification of Okinawan vegetable consumption. In addition, it is uncertain whether misclassification resulted in biased associations toward or away from the null. Finally, although we measured and adjusted for possible confounding variables as much as possible, there could still be potential residual confounding by unmeasured variables.

In conclusion, this prospective cohort study revealed that total Okinawan vegetable consumption and specific Okinawan vegetable consumption were not statistically significantly associated with risk of incident stroke and coronary heart disease in the Japanese population of Okinawa Prefecture.

## APPENDIX A. SUPPLEMENTARY DATA

eTable 1. Names of 7 Okinawan vegetables, 15 fruits, and 24 vegetables assessed using a validated comprehensive food frequency questionnaire

eTable 2. Dietary information of Okinawan vegetables in the Standard Tables of Food Composition in Japan - 2015 (Seventh Revised Version)

eTable 3. Age- and study area-adjusted and multivariate-adjusted hazard ratios and 95% confidence intervals of incident cardiovascular outcomes according to tertiles of consumption of total vegetables and fruits, total vegetables, and total fruits in men and women

eTable 4. Age- and study area-adjusted and multivariate-adjusted hazard ratios and 95% confidence intervals of incident cardiovascular outcomes according to tertiles of total and specific Okinawan vegetable consumption in men and women
