# Supplementary material for: Association of Vegetable, Fruit, and Okinawan Vegetable Consumption With Incident Stroke and Coronary Heart Disease
Source: J Epidemiol. 2020 Jan 5;30(1):37–45. doi: 10.2188/jea.JE20180130 (PMC6908839; doi:10.2188/jea.JE20180130)
Supplement: Supplementary file 1 [file je-30-037-s001.pdf]

eTable 1. Names of 7 Okinawan vegetables, 15 fruits, and 24 vegetables assessed using a validated comprehensive food frequency questionnaire

Total vegetables and fruits				
Total vegetables			Fruits	
Vegetables		Okinawan vegetables		
Chinese radish	Chinese chives	Pak choi	Mandarin orange	Watermelon
Green leafy vegetables	Garland chrysanthemums	Leaf mustard	Other oranges	Peach
Plum	Green pepper	Bitter gourd	100% orange juice	Pear
Chinese cabbage	Carrot	Swiss chard	Apple	Kiwi fruit
Cucumber	Tomato	Loofah	Persimmon	Pineapple
Eggplant	Pumpkin	Mugwort	Strawberry	Banana
Cabbage	Tomato juice	Papaya	Grapes	100% apple juice
Chinese radish	Onion		Melon	
Komatsuna	Cucumber			
Broccoli	Bean sprouts			
Chinese cabbage	Snap beans			
Spinach	Lettuce			

eTable 2. Dietary information of Okinawan vegetables in the Standard Tables of Food Composition in Japan - 2015 (Seventh Revised Version)

		Okinawan vegetables						
		Pak choi	Leaf mustard	Bitter gourd	Swiss chard	Loofah	Mugwort	Papaya[a]
Energy	kcal/100 g	9	26	17	19	16	46	39
Water	g/100 g	96	90.3	94.4	92.2	94.9	83.6	88.7
Protein	g/100 g	0.6	3.3	1	2	0.8	5.2	1.3
Fat	g/100 g	0.1	0.1	0.1	0.1	0.1	0.3	0.1
Carbohydrate	g/100 g	2	4.7	3.9	3.7	3.8	8.7	9.4
K	mg/100 g	260	620	260	1200	150	890	190
Ca	mg/100 g	100	140	14	75	12	180	36
Mg	mg/100 g	16	21	14	74	12	29	19
Fe	mg/100 g	1.1	2.2	0.4	3.6	0.3	4.3	0.3
Zinc	mg/100 g	0.3	0.9	0.2	0.3	0.2	0.6	0.1
Vitamin A	µg/100 g	170	230	17	310	4	440	10
Vitamin D	µg/100 g	0	0	0	0	0	0	0
Vitamin E	mg/100 g	0.7	3	0.8	1.7	0.3	3.2	0.1
Vitamin K	µg/100 g	84	260	41	180	12	340	0
Vitamin B1	mg/100 g	0.03	0.12	0.05	0.07	0.03	0.19	0.03
Vitamin B2	mg/100 g	0.07	0.27	0.07	0.23	0.04	0.34	0.04
Vitamin B6	mg/100 g	0.08	0.25	0.06	0.25	0.07	0.08	0.01
Vitamin B12	µg/100 g	0	0	0	0	0	0	0
Folate	µg/100 g	66	310	72	120	92	190	38
Vitamin C	mg/100 g	24	64	76	19	5	35	45
Total fibre	g/100 g	1.2	3.7	2.6	3.3	1	7.8	2.2

[a]Data were based on the dietary information of green papaya (immature papaya).

eTable 3. Age- and study area-adjusted and multivariate-adjusted hazard ratios and 95% confidence intervals of incident cardiovascular outcomes according to tertiles of consumption of total vegetables and fruits, total vegetables, and total fruits in men and women

		Lowest (T1)	Middle (T2)		Highest (T3)		*p for trend*	Lowest (T1)	Middle (T2)		Highest (T3)		*p for trend*
		Ref.	HR	(95% CI)	HR	(95% CI)		Ref.	HR	(95% CI)	HR	(95% CI)	
**Total vegetable and fruit consumption**[e]		Men						Women					
Number of participants		2,575	2,576		2,575		-	2,924	2,924		2,924		-
Median intake, g/day		164.5	305.3		502.5		-	213.1	367.3		574.1		-
Person-years		32,747	33,218		32,954		-	39,405	39,582		39,560		-
Cardiovascular disease, total	Number of cases	215	198		209			136	133		145		
	Model 1[a]	1.0	0.84	(0.69 - 1.01)	0.86	(0.71 - 1.04)	0.119	1.0	0.92	(0.73 - 1.17)	0.96	(0.76 - 1.21)	0.737
	Model 2[b]	1.0	0.91	(0.74 - 1.11)	0.99	(0.80 - 1.23)	0.949	1.0	1.00	(0.78 - 1.29)	1.09	(0.84 - 1.40)	0.529
	Model 3[c]	1.0	0.91	(0.75 - 1.12)	0.99	(0.80 - 1.23)	0.910	1.0	1.01	(0.79 - 1.30)	1.06	(0.82 - 1.38)	0.640
Stroke, total	Number of cases	171	148		156			120	119		125		
	Model 1[a]	1.0	0.79	(0.63 - 0.98)	0.81	(0.65 - 1.00)	0.053	1.0	0.94	(0.73 - 1.21)	0.94	(0.73 - 1.21)	0.621
	Model 2[b]	1.0	0.86	(0.68 - 1.08)	0.94	(0.74 - 1.21)	0.631	1.0	1.02	(0.79 - 1.33)	1.06	(0.81 - 1.40)	0.680
	Model 3[c]	1.0	0.87	(0.69 - 1.10)	0.94	(0.74 - 1.21)	0.626	1.0	1.03	(0.79 - 1.34)	1.04	(0.79 - 1.37)	0.799
Intraparenchymal haemorrha	Number of cases	58	50		51			43	42		47		
	Model 1[a]	1.0	0.81	(0.55 - 1.18)	0.81	(0.55 - 1.18)	0.265	1.0	0.93	(0.61 - 1.42)	0.99	(0.65 - 1.50)	0.970
	Model 2[b]	1.0	0.94	(0.63 - 1.40)	1.01	(0.66 - 1.55)	0.975	1.0	1.05	(0.68 - 1.63)	1.22	(0.78 - 1.92)	0.386
	Model 3[c]	1.0	0.95	(0.64 - 1.42)	1.01	(0.66 - 1.56)	0.961	1.0	1.05	(0.67 - 1.63)	1.20	(0.77 - 1.90)	0.420
Ischaemic stroke	Number of cases	103	96		99			66	60		62		
	Model 1[a]	1.0	0.83	(0.63 - 1.09)	0.82	(0.62 - 1.09)	0.174	1.0	0.85	(0.60 - 1.21)	0.83	(0.59 - 1.18)	0.303
	Model 2[b]	1.0	0.88	(0.66 - 1.17)	0.93	(0.69 - 1.27)	0.658	1.0	0.90	(0.62 - 1.29)	0.85	(0.58 - 1.24)	0.390
	Model 3[c]	1.0	0.89	(0.67 - 1.19)	0.93	(0.68 - 1.27)	0.633	1.0	0.91	(0.63 - 1.31)	0.82	(0.56 - 1.20)	0.305
Coronary heart disease (myocardial infarction or sudden cardiac death)	Number of cases	44	50		53			16	14		20		
	Model 1[a]	1.0	1.02	(0.68 - 1.53)	1.06	(0.71 - 1.58)	0.778	1.0	0.82	(0.40 - 1.68)	1.12	(0.58 - 2.15)	0.716
	Model 2[b]	1.0	1.09	(0.71 - 1.65)	1.20	(0.77 - 1.87)	0.416	1.0	0.86	(0.41 - 1.81)	1.24	(0.60 - 2.58)	0.542
	Model 3[c]	1.0	1.07	(0.70 - 1.63)	1.17	(0.75 - 1.83)	0.482	1.0	0.88	(0.42 - 1.85)	1.27	(0.61 - 2.63)	0.510
**Total vegetable consumption**[e]													
Number of participants		2,575	2,576		2,575		-	2,924	2,924		2,924		-
Median intake, g/day		98.8	187.1		319.9		-	121.4	217.1		353.7		-
Person-years		32,945	33,089		32,885		-	39,404	39,673		39,471		-
Cardiovascular disease, total	Number of cases	199	207		216			140	119		155		
	Model 1[a]	1.0	0.99	(0.82 - 1.21)	0.97	(0.80 - 1.18)	0.745	1.0	0.80	(0.63 - 1.03)	0.96	(0.76 - 1.21)	0.775
	Model 2[b, d]	1.0	1.11	(0.91 - 1.37)	1.18	(0.94 - 1.47)	0.151	1.0	0.89	(0.69 - 1.14)	1.12	(0.86 - 1.46)	0.390
	Model 3[c]	1.0	1.10	(0.89 - 1.35)	1.15	(0.92 - 1.44)	0.230	1.0	0.88	(0.68 - 1.13)	1.08	(0.83 - 1.41)	0.553
Stroke, total	Number of cases	156	152		167			126	101		137		
	Model 1[a]	1.0	0.94	(0.75 - 1.17)	0.96	(0.77 - 1.20)	0.720	1.0	**0.76**	**(0.59 - 0.99)**	0.95	(0.74 - 1.21)	0.711
	Model 2[b, d]	1.0	1.08	(0.86 - 1.37)	1.23	(0.96 - 1.59)	0.106	1.0	0.84	(0.64 - 1.10)	1.10	(0.83 - 1.46)	0.486
	Model 3[c]	1.0	1.07	(0.84 - 1.35)	1.20	(0.92 - 1.54)	0.174	1.0	0.83	(0.63 - 1.09)	1.07	(0.81 - 1.41)	0.639
Intraparenchymal haemorrha	Number of cases	47	53		59			47	36		49		
	Model 1[a]	1.0	1.12	(0.75 - 1.65)	1.17	(0.80 - 1.73)	0.417	1.0	0.73	(0.47 - 1.13)	0.92	(0.62 - 1.37)	0.704
	Model 2[b, d]	1.0	1.48	(0.97 - 2.24)	**1.82**	**(1.15 - 2.86)**	**0.010**	1.0	0.83	(0.53 - 1.30)	1.14	(0.72 - 1.82)	0.570
	Model 3[c]	1.0	1.47	(0.97 - 2.22)	**1.78**	**(1.13 - 2.80)**	**0.013**	1.0	0.82	(0.52 - 1.30)	1.13	(0.71 - 1.80)	0.592
Ischaemic stroke	Number of cases	102	92		104			65	53		70		
	Model 1[a]	1.0	0.85	(0.64 - 1.13)	0.89	(0.67 - 1.17)	0.394	1.0	0.77	(0.53 - 1.10)	0.92	(0.65 - 1.29)	0.646
	Model 2[b, d]	1.0	0.93	(0.69 - 1.24)	1.05	(0.77 - 1.44)	0.758	1.0	0.83	(0.57 - 1.22)	0.99	(0.67 - 1.46)	0.977
	Model 3[c]	1.0	0.91	(0.67 - 1.22)	1.01	(0.73 - 1.39)	0.946	1.0	0.82	(0.56 - 1.20)	0.94	(0.63 - 1.39)	0.758
Coronary heart disease (myocardial infarction or sudden cardiac death)	Number of cases	43	55		49			14	18		18		
	Model 1[a]	1.0	1.18	(0.79 - 1.76)	1.01	(0.67 - 1.52)	0.993	1.0	1.18	(0.59 - 2.37)	1.07	(0.53 - 2.16)	0.866
	Model 2[b, d]	1.0	1.21	(0.80 - 1.84)	1.04	(0.66 - 1.66)	0.876	1.0	1.29	(0.62 - 2.69)	1.25	(0.56 - 2.81)	0.598
	Model 3[c]	1.0	1.18	(0.78 - 1.80)	1.04	(0.65 - 1.66)	0.881	1.0	1.25	(0.60 - 2.61)	1.18	(0.53 - 2.66)	0.695
**Total fruit consumption**[e]													
Number of participants		2,575	2,576		2,575		-	2,924	2,924		2,924		-
Median intake, g/day		30.3	102.0		208.5		-	49.7	130.9		247.6		-
Person-years		32,820	33,026		33,072		-	39,284	39,984		39,279		-
Cardiovascular disease, total	Number of cases	223	203		196			150	124		140		
	Model 1[a]	1.0	0.88	(0.73 - 1.06)	0.82	(0.68 - 1.00)	**0.045**	1.0	0.84	(0.66 - 1.06)	0.97	(0.77 - 1.22)	0.756
	Model 2[b, d]	1.0	0.94	(0.77 - 1.14)	0.88	(0.72 - 1.08)	0.228	1.0	0.87	(0.68 - 1.11)	1.01	(0.79 - 1.29)	0.926
	Model 3[c]	1.0	0.95	(0.78 - 1.16)	0.91	(0.74 - 1.11)	0.344	1.0	0.89	(0.70 - 1.14)	1.03	(0.81 - 1.31)	0.829
Stroke, total	Number of cases	174	159		142			133	114		117		
	Model 1[a]	1.0	0.88	(0.71 - 1.09)	**0.76**	**(0.61 - 0.95)**	**0.017**	1.0	0.87	(0.67 - 1.11)	0.91	(0.71 - 1.16)	0.430
	Model 2[b, d]	1.0	0.95	(0.76 - 1.19)	0.83	(0.65 - 1.05)	0.119	1.0	0.90	(0.70 - 1.17)	0.97	(0.74 - 1.26)	0.803
	Model 3[c]	1.0	0.97	(0.77 - 1.21)	0.86	(0.67 - 1.09)	0.207	1.0	0.92	(0.71 - 1.20)	0.98	(0.75 - 1.28)	0.871
Intraparenchymal haemorrha	Number of cases	64	51		44			48	37		47		
	Model 1[a]	1.0	0.77	(0.54 - 1.12)	**0.66**	**(0.45 - 0.96)**	**0.029**	1.0	0.78	(0.51 - 1.19)	1.00	(0.67 - 1.50)	0.998
	Model 2[b, d]	1.0	0.80	(0.55 - 1.18)	0.67	(0.44 - 1.02)	0.061	1.0	0.77	(0.50 - 1.21)	1.07	(0.70 - 1.65)	0.745
	Model 3[c]	1.0	0.81	(0.55 - 1.19)	0.69	(0.45 - 1.04)	0.075	1.0	0.78	(0.50 - 1.22)	1.07	(0.69 - 1.64)	0.770
Ischaemic stroke	Number of cases	103	103		92			74	62		52		
	Model 1[a]	1.0	0.96	(0.73 - 1.26)	0.82	(0.62 - 1.09)	0.178	1.0	0.85	(0.61 - 1.19)	0.73	(0.51 - 1.04)	0.082
	Model 2[b, d]	1.0	1.05	(0.79 - 1.39)	0.89	(0.66 - 1.21)	0.466	1.0	0.92	(0.65 - 1.30)	0.77	(0.53 - 1.12)	0.181
	Model 3[c]	1.0	1.07	(0.80 - 1.42)	0.93	(0.68 - 1.26)	0.631	1.0	0.95	(0.67 - 1.35)	0.79	(0.54 - 1.15)	0.230
Coronary heart disease (myocardial infarction or sudden cardiac death)	Number of cases	49	44		54			17	10		23		
	Model 1[a]	1.0	0.88	(0.58 - 1.32)	1.03	(0.70 - 1.52)	0.861	1.0	0.61	(0.28 - 1.33)	1.45	(0.78 - 2.72)	0.216
	Model 2[b, d]	1.0	0.91	(0.60 - 1.38)	1.09	(0.72 - 1.65)	0.655	1.0	0.61	(0.27 - 1.36)	1.30	(0.67 - 2.53)	0.356
	Model 3[c]	1.0	0.90	(0.59 - 1.37)	1.08	(0.71 - 1.63)	0.719	1.0	0.64	(0.28 - 1.43)	1.37	(0.71 - 2.68)	0.292

CI, confidence interval; HR, hazard ratio.

[a]Model 1 was adjusted by age and study area.

[b]Model 2 was adjusted by variables in model 1 plus alcohol intake (0, 1–150, 151–300, 301–450, and ≥451 g/week, or missing), cigarette smoking status (current [<20 or ≥20 cigarettes/day], never, former, or missing), type of work (blue-collar, white-collar, other, or missing), self-reported perceived mental stress (low, medium, high, or missing), quartiles of body mass index, metabolic equivalent task-hours per day, quartiles of energy intake, and energy-adjusted dietary consumption of fish, meat, and sodium.

[c]Model 3 was adjusted by variables in model 2 plus past history of diabetes (yes, no, or missing), treatment of hypertension (yes, no, or missing), and treatment of hypercholesterolaemia (yes, no, or missing).

[d]Quartiles of energy-adjusted dietary consumption of total fruits were added to model 2 in the multivariate analysis for total vegetables, and quartiles of total vegetable consumption were added for total fruits.

[e]Vegetable and fruit consumption was energy-adjusted by the nutrient residual method.

eTable 4. Age- and study area-adjusted and multivariate-adjusted hazard ratios and 95% confidence intervals of incident cardiovascular outcomes according to tertiles of total and specific Okinawan vegetable consumption in men and women

			Lowest (T1)	Middle (T2)		Highest (T3)		*p for trend*	Lowest (T1)	Middle (T2)		Highest (T3)		*p for trend*
			Ref.	HR	(95% CI)	HR	(95% CI)		Ref.	HR	(95% CI)	HR	(95% CI)	
**Total Okinawan vegetable consumption[d]**						Men						Women		
	Number of participants		2,575	2,576		2,575		-	2,924	2,924		2,924		-
	Median intake, g/day		15.8	40.3		89.4		-	20.0	47.5		98.4		-
	Person-years		32,760	33,044		33,116		-	39,387	39,671		39,490		-
	Cardiovascular disease, total	Number of cases	210	187		225			143	123		148		
		Model 1[a]	1.0	0.84	(0.69 - 1.02)	0.97	(0.80 - 1.17)	0.780	1.0	0.83	(0.65 - 1.06)	0.95	(0.76 - 1.20)	0.691
		Model 2[b]	1.0	0.89	(0.72 - 1.09)	1.06	(0.86 - 1.32)	0.557	1.0	0.88	(0.69 - 1.13)	1.05	(0.81 - 1.36)	0.713
		Model 3[c]	1.0	0.88	(0.72 - 1.08)	1.05	(0.85 - 1.30)	0.652	1.0	0.88	(0.68 - 1.13)	1.03	(0.79 - 1.33)	0.838
	Stroke, total	Number of cases	162	139		174			123	110		131		
		Model 1[a]	1.0	0.82	(0.65 - 1.02)	0.99	(0.80 - 1.23)	0.961	1.0	0.87	(0.67 - 1.12)	0.99	(0.77 - 1.27)	0.963
		Model 2[b]	1.0	0.89	(0.70 - 1.13)	1.12	(0.88 - 1.44)	0.352	1.0	0.94	(0.72 - 1.23)	1.13	(0.85 - 1.50)	0.390
		Model 3[c]	1.0	0.88	(0.69 - 1.11)	1.09	(0.85 - 1.40)	0.478	1.0	0.94	(0.72 - 1.22)	1.11	(0.84 - 1.47)	0.464
	Intraparenchymal haemorr	Number of cases	52	48		59			48	37		47		
		Model 1[a]	1.0	0.90	(0.61 - 1.33)	1.09	(0.75 - 1.59)	0.628	1.0	0.75	(0.49 - 1.16)	0.93	(0.62 - 1.39)	0.710
		Model 2[b]	1.0	1.06	(0.70 - 1.60)	1.29	(0.84 - 2.00)	0.247	1.0	0.80	(0.51 - 1.25)	1.05	(0.66 - 1.65)	0.864
		Model 3[c]	1.0	1.06	(0.70 - 1.61)	1.28	(0.83 - 1.99)	0.262	1.0	0.81	(0.52 - 1.26)	1.04	(0.66 - 1.64)	0.884
	Ischaemic stroke	Number of cases	103	84		111			62	60		66		
		Model 1[a]	1.0	0.76	(0.57 - 1.02)	0.96	(0.73 - 1.26)	0.817	1.0	0.93	(0.65 - 1.33)	0.97	(0.69 - 1.38)	0.888
		Model 2[b]	1.0	0.79	(0.59 - 1.07)	1.06	(0.78 - 1.45)	0.678	1.0	1.04	(0.72 - 1.51)	1.14	(0.77 - 1.69)	0.526
		Model 3[c]	1.0	0.78	(0.58 - 1.06)	1.03	(0.75 - 1.40)	0.855	1.0	1.03	(0.71 - 1.49)	1.09	(0.73 - 1.62)	0.667
	Coronary heart disease (myocardial infarction or sudden cardiac death)	Number of cases	48	48		51			20	13		17		
		Model 1[a]	1.0	0.92	(0.61 - 1.37)	0.91	(0.61 - 1.35)	0.633	1.0	0.60	(0.30 - 1.21)	0.71	(0.37 - 1.36)	0.305
		Model 2[b]	1.0	0.89	(0.58 - 1.35)	0.91	(0.58 - 1.41)	0.666	1.0	0.60	(0.29 - 1.24)	0.68	(0.33 - 1.42)	0.301
		Model 3[c]	1.0	0.91	(0.60 - 1.38)	0.93	(0.60 - 1.45)	0.766	1.0	0.60	(0.29 - 1.24)	0.67	(0.32 - 1.38)	0.265
**Specific Okinawan vegetable consumption[d]**														
Pak choi	Median intake, g/day		0.0	2.4		9.8			0.0	2.9		13.0		
	Cardiovascular disease, total	Model 3[c]	1.0	1.21	(0.99 - 1.48)	1.05	(0.85 - 1.29)	0.591	1.0	0.90	(0.71 - 1.15)	1.00	(0.78 - 1.29)	0.973
Leaf musta	Median intake, g/day		0.0	2.5		9.8			0.0	2.8		11.3		
	Cardiovascular disease, total	Model 3[c]	1.0	**1.28**	**(1.05 - 1.56)**	1.09	(0.88 - 1.36)	0.402	1.0	1.02	(0.80 - 1.30)	1.00	(0.77 - 1.30)	0.985
Bitter gou	Median intake, g/day		4.2	14.8		38.6			4.7	16.3		39.8		
	Cardiovascular disease, total	Model 3[c]	1.0	0.89	(0.73 - 1.10)	1.03	(0.84 - 1.27)	0.774	1.0	0.85	(0.67 - 1.09)	0.90	(0.70 - 1.16)	0.393
Swiss cha	Median intake, g/day		0.0	1.6		4.6			0.0	1.9		5.8		
	Cardiovascular disease, total	Model 3[c]	1.0	0.93	(0.62 - 1.39)	1.09	(0.91 - 1.29)	0.364	1.0	1.21	(0.86 - 1.71)	1.04	(0.84 - 1.29)	0.678
Loofah	Median intake, g/day		1.5	4.9		20.6			1.8	6.2		23.3		
	Cardiovascular disease, total	Model 3[c]	1.0	1.21	(0.99 - 1.48)	1.20	(0.97 - 1.47)	0.095	1.0	0.80	(0.62 - 1.02)	0.98	(0.76 - 1.25)	0.836
Mugwort	Median intake, g/day		0.0	0.2		0.4			0.0	0.2		0.5		
	Cardiovascular disease, total	Model 3[c]	1.0	1.02	(0.83 - 1.25)	1.04	(0.86 - 1.27)	0.667	1.0	1.07	(0.83 - 1.37)	1.03	(0.81 - 1.32)	0.817
Papaya	Median intake, g/day		0.0	3.2		9.8			1.2	3.5		11.3		
	Cardiovascular disease, total	Model 3[c]	1.0	0.97	(0.80 - 1.19)	1.02	(0.83 - 1.24)	0.869	1.0	0.96	(0.75 - 1.24)	1.12	(0.88 - 1.43)	0.341

CI, confidence interval; HR, hazard ratio.

[a]Model 1 was adjusted by age and study area.

[b]Model 2 was adjusted by variables in model 1 plus alcohol intake (0, 1–150, 151–300, 301–450, and ≥451 g/week, or missing), cigarette smoking status (current [<20 or ≥20 cigarettes/day], never, former, or missing), type of work (blue-collar, white-collar, other, or missing), self-reported perceived mental stress (low, medium, high, or missing), quartiles of body mass index, metabolic equivalent task-hours per day, quartiles of energy intake, and energy-adjusted dietary consumption of fish, meat, vegetable (except for Okinawan vegetables), fruit (except for papaya), and sodium.

[c]Model 3 was adjusted by variables in model 2 plus past history of diabetes (yes, no, or missing), treatment of hypertension (yes, no, or missing), and treatment of hypercholesterolaemia (yes, no, or missing).

[d]Total and specific Okinawan vegetable consumption was energy-adjusted using the nutrient residual method.
